# Molluscan RXR Transcriptional Regulation by Retinoids in a *Drosophila* CNS Organ Culture System

**DOI:** 10.3390/cells11162493

**Published:** 2022-08-11

**Authors:** Eric de Hoog, Victoria Elda Saba Echezarreta, Anel Turgambayeva, Gregory Foran, Marvel Megaly, Aleksandar Necakov, Gaynor E. Spencer

**Affiliations:** Department of Biological Sciences, Brock University, St. Catharines, ON L2S 3A1, Canada

**Keywords:** retinoids, nervous system, all-*trans* retinoic acid, 9-*cis* retinoic acid, *Lymnaea stagnalis*, EC23, SR11237

## Abstract

Retinoic acid, the active metabolite of Vitamin A, is important for the appropriate development of the nervous system (e.g., neurite outgrowth) as well as for cognition (e.g., memory formation) in the adult brain. We have shown that many of the effects of retinoids are conserved in the CNS of the mollusc, *Lymnaea stagnalis*. RXRs are predominantly nuclear receptors, but the *Lymnaea* RXR (LymRXR) exhibits a non-nuclear distribution in the adult CNS, where it is also implicated in non-genomic retinoid functions. As such, we developed a CNS *Drosophila* organ culture-based system to examine the transcriptional activity and ligand-binding properties of LymRXR, in the context of a live invertebrate nervous system. The novel ligand sensor system was capable of reporting both the expression and transcriptional activity of the sensor. Our results indicate that the LymRXR ligand sensor mediated transcription following activation by both 9-*cis* RA (the high affinity ligand for vertebrate RXRs) as well as the vertebrate RXR synthetic agonist, SR11237. The LymRXR ligand sensor was also activated by all-*trans* RA, and to a much lesser extent by the vertebrate RAR synthetic agonist, EC23. This sensor also detected endogenous retinoid-like activity in the CNS of developing *Drosophila* larvae, primarily during the 3^rd^ instar larval stage. These data indicate that the LymRXR sensor can be utilized not only for characterization of ligand activation for studies related to the *Lymnaea* CNS, but also for future studies of retinoids and their functions in *Drosophila* development.

## 1. Introduction

Retinoid receptors belong to the large superfamily of nuclear receptors (NRs), one of the largest groups of ligand-activated transcription factors which play important roles in endocrine processes. There are two main families of retinoid receptors, the retinoic acid receptors (RARs) and the retinoid X receptors (RXRs) for which, at least in vertebrates, there are three different subtypes (α, β, and γ), with various splice variants. The RXRs are found in species from jellyfish [1] to humans [2] and are implicated as promising therapeutic targets in many diseases, including cancer and neurodegenerative diseases such as Alzheimer’s and multiple sclerosis [3].

The retinoid receptors bind the small lipophilic metabolite of Vitamin A, retinoic acid (RA), for which at least two main isomers, all-*trans* RA and 9-*cis* RA, exist. In vertebrates, RXRs can act as heterodimerization partners with the RAR, as well as many other NRs, and binding studies suggest that vertebrate RXRs preferentially bind the 9-*cis* RA isomer [4]. However, the presence of this isomer in mammalian tissues has not been clearly demonstrated; it is speculated that it may be a product of all-*trans* RA isomerization, and its role as the endogenous RXR ligand has been questioned. More recently, 9-*cis*-13,14-dihydroretinoic acid was shown to bind to and transactivate RXR and higher endogenous levels of this retinoid have led to the proposal that it might be the endogenous RXR ligand, at least in mice [5].

*RXR* is considered an ancient gene that is essential for animal development and homeostasis [6] and analyses of full-length *RXR* gene orthologs in a number of metazoan species suggest they are largely conserved. RXRs are exploited by endocrine-disrupting chemicals which can cause physiological changes in development, metabolism and reproduction. As such, molluscan RXRs have been examined in several aquatic species in the context of monitoring endocrine disruption as a consequence of ecological toxicity [7,8,9,10]. Though several studies have shown that RXR ligands (including organotins such as tributyltin [TBT]) can induce imposex in gastropod molluscs, few studies have examined the effects of retinoids in the molluscan CNS. Our studies using the pond snail, *Lymnaea stagnalis*, have shown conserved effects of retinoids in the molluscan nervous system, which include induction of neurite outgrowth [11], axon pathfinding [12], synaptic function [13], learning and memory [14], as well as novel roles in ion channel modulation [15,16,17]. Though invertebrate, non-chordate homologues of the RAR have now been discovered [18,19], RARs from only two (non-molluscan) species have been shown to bind retinoids to date (*Platynereis dumerilii* [20] and *Priapulus caudatus* [21]). As such, it is possible that many of the physiological effects of retinoids in invertebrates may involve activation of the RXR. 

Molluscan RXRs show high sequence homology to vertebrate RXRα [22,23], and have been shown to bind 9-*cis* RA and to transactivate transcription in species such as *B. glabrata* [22], *N. Lapillus* [7] and *T. clavigera* [10]. In the CNS of *Lymnaea stagnalis*, the RXR protein exhibits a non-nuclear cellular localization, present in the neuropil, neurites and growth cones [23]. LymRXR was only found to be present in the nuclear fraction in embryos. Despite the lack of apparent nuclear localization in the adult CNS, both all-*trans* and 9-*cis* RA isomers induce neurite outgrowth and exert chemotropic effects, as they do in vertebrates. Many of these effects are also mimicked by vertebrate RXR agonists and/or inhibited by vertebrate RXR antagonists. However, a number of retinoid effects in the adult *Lymnaea* CNS are rapid and non-genomic in nature and to date, we have not yet determined whether the *Lymnaea* RXR can regulate transcription (as it does in most other molluscan and vertebrate species), nor determined which retinoid isomers it can bind. 

In this study, we designed a novel ligand sensor using the *Lymnaea* RXR ligand binding domain (LBD), and developed a live *Drosophila* CNS organ culture system, in which we were able to simultaneously quantify both the expression of the ligand sensor as well as its transcriptional activity. Our results indicate both similarities and differences in ligand activation of the LymRXR compared to vertebrate RXRs, but also provide evidence for endogenous retinoid-like activity in *Drosophila* larvae.

## 2. Materials and Methods

### 2.1. LymRXR Ligand Sensor Construct

**Figure 1:**
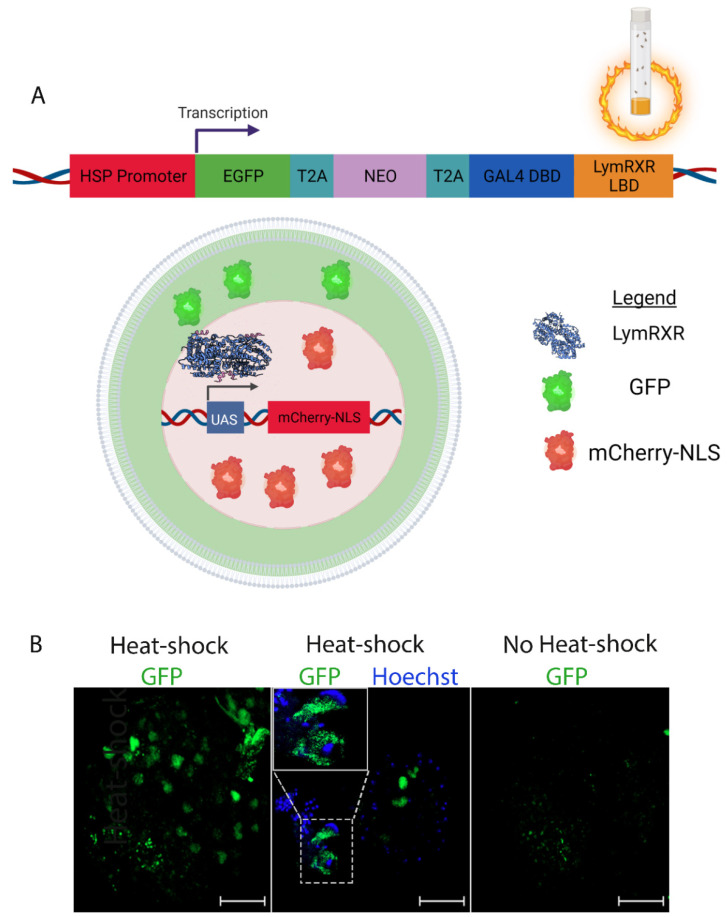
Ligand sensor design and expression. (**A**) Design of the ligand sensor system. Heat-shock of LymRXR ligand sensor flies results in cellular expression of GFP (primarily cytoplasmic localization) and the LymRXR ligand sensor (nuclear localization). Upon activation of LymRXR sensor while bound to UAS sequences, there is expression of nuclear localized mCherry (image made with Biorender). (**B**) Representative images of GFP expression in the CNS of LymRXR sensor larvae (fly line #2) following heat-shock (left and middle panels), compared to non-heat-shocked controls (right panel). The middle panel shows both GFP expression and Hoechst nuclear stain in a surface image of a CNS hemisphere. Inset shows enlarged image of cells showing clear cytoplasmic localization of GFP, with minimal overlap with Hoechst. Scale bars: 50 μm.

We designed a novel ligand sensor system that consists of 5 distinct components (Figure 1A): 1. eGFP 2. T2A 3. a transgene that confers neomycin (Neo) resistance 4. a T2A autocleavable peptide sequence carrying degenerate codons (dT2A) and 5. a GAL4 DNA-binding domain with an N-terminal FLAG-tag fused to the N-terminus of the hinge and ligand-binding domain (LBD) of the *Lymnaea stagnalis* RXR (LymRXR) (for complete sequence see Figure S1). This ligand sensor construct was generated by gene synthesis (Biobasic) and inserted into the PRExpress vector backbone [24]. PRExpress was a gift from Renato Paro (plasmid # 122486; http://n2t.net/addgene:122486; RRID:Addgene_122486, accessed on 13 June 2019). Expression of this novel ligand sensor system was under the control of the heat shock protein 70 (HSP70) promoter, and flanked by gypsy insulator sites, which eliminate leaky transgene expression by blocking non-specific transcriptional activation by adjacent genomic regulatory sequences [24]. This ligand sensor expression construct also contained an attB integration sequence, which allowed for site-specific genomic integration of each ligand sensor construct into the attP2 site on chromosome 3 of the *Drosophila genome* using PhiC31 integrase (Best Gene; BDSC stock #:8622). The Neomycin cassette was included for improved selection for future cell culture purposes, and the FLAG-tag to provide compatibility for future immunostaining or western blotting. 

### 2.2. Fly Maintenance and Genetics

*Drosophila melanogaster* fly stocks were reared on standard German food (Bloomington Drosophila Stock Centre [BDSC]) in standard acrylic fly vials and kept at room temperature (20 °C ± 1 °C). The fly lines used in this study were as follows:

1. w; +/+; UAS-mCherry-NLS. Expression of nuclear-localized mCherry under the control of the UAS promoter. (Bloomington Stock #: 38424).

2. w; +/+; HSP-eGFP-T2A-Neo-T2A-LymRXR sensor/TM3. Expression of eGFP and LymRXR sensor under the control of the HSP70 promoter.

3. w; +/+; HSP-eGFP-T2A-Neo-T2A-LymRXR sensor/UAS-mCherry-NLS. Expression of eGFP and LymRXR sensor under the control of the HSP70 promoter and nuclear localized mCherry under the control of the UAS promoter.

Homozygous UAS-mCherry-NLS virgin females were crossed to heterozygous *Lym*RXR males (#2 fly line did not exhibit homozygotes). Both foraging (feeding, non-crawling), and wandering (non-feeding, wall crawling) 3rd instar larvae [25] were used for experiments. For all experiments (unless otherwise stated), 3rd instar larvae (either foraging or wandering) were heat-shocked at 37 °C for one hour and transferred to a culture dish containing 1X PBS. Larvae were then dissected in complete Schneider’s media (Schneider’s insect media containing 10% FBS, 1% Penicillin-Streptomycin and 0.1% insulin) and their CNS removed and placed in complete Schneider’s media. CNS preparations were then incubated in either DMSO (vehicle control) or a retinoid for 24 h at 21 °C, prior to live imaging and those exhibiting sensor expression were used for analysis. 

To study in vivo LymRXR sensor activation during *Drosophila* development (in the absence of exogenous application of retinoids), LymRXR sensor embryos (fly line #3) were first collected for 5 to 6 h, and subsequently heat-shocked throughout development for 1 h, every 24 h, until they reached the wandering 3rd instar larval stage. To investigate at which stage endogenous sensor activation occurred (in the absence of exogenously applied retinoids), heat-shock treatment of progeny was also initiated at different developmental stages: after embryogenesis, after the 1st larval stage (L1), or after the 2nd larval stage (L2). This experimental design corresponded to progeny not being heat-shocked for approximately 24, 48, or 72 h after embryo collection, respectively. CNS from heat-shocked larvae were dissected once they reached the wandering 3rd instar larval stage and cultured at 21 °C for 24 h (but in the absence of any retinoid treatment), prior to live imaging, and those exhibiting sensor expression were used for analysis. As controls, we utilized CNS from LymRXR sensor larvae (wandering 3rd instar) that were not heat-shocked during their development.

### 2.3. Chemicals

All-*trans* and 9-*cis* retinoic acid were obtained from Sigma whereas synthetic retinoids, SR11237 and EC23 were obtained from Tocris Bioscience. Stock solutions of retinoids were made as a 10^−2^ M stock in 100% DMSO. All-*trans* retinoic acid was used at a final bath concentration of either 10, 5 or 1 μM. 9-*cis* retinoic acid, SR11237, and EC23 were used at a final bath concentration of 1 μM. Control experiments were performed using an equivalent concentration of DMSO. 

### 2.4. Imaging

Hemispheres of the larval CNS were imaged live in complete Schneider’s media on an inverted Zeiss Axio Observer spinning disc confocal microscope equipped with a Yokogawa spinning disc head and a Prime BSI 16-bit camera. Prior to imaging, cultured larval CNS were incubated in Hoechst (33342; 1:1000 dilution) for 20–30 min (to provide a nuclear stain). 0.44 μm optical sections of one or both CNS hemispheres were taken with a 20× Plan Apochromat 0.8 NA objective using 350–400 nm, 450–490 nm, and 545–575 nm laser lines and 422 nm, 517 nm, and 603 nm filters for Hoechst, GFP, and mCherry, respectively. A minimum of 3 experimental replicates (each with multiple CNS preparations), were conducted for each retinoid or vehicle treatment. Within each replicate, all retinoid and vehicle-treated CNS were imaged on the same day. For the in vivo ligand sensor activation experiments during *Drosophila* development, a minimum of three replicates were performed.

### 2.5. Image Analysis

In this study, we quantified the relative number of cells producing our ligand sensor, which was represented as the number of GFP “punctae”. We also simultaneously quantified the relative number of mCherry-expressing cells, representing ligand sensor reporter expression in the nuclei, and represented herein as the number of mCherry “punctae” per hemisphere, (where one punctae is representative of approximately one nucleus). That is, an increase in the number of mCherry punctae corresponded to an increase in the number of cells containing mCherry. We also quantified the proportion of GFP punctae that contained mCherry-expressing nuclei (GFP Manders coefficient), and the proportion of mCherry nuclei that contained GFP (mCherry Manders coefficient). The integrated density of GFP and mCherry was defined as the mean fluorescence intensity per puncta x the total area of the punctae (in microns). To analyze these parameters, we generated an image analysis pipeline in FIJI [26]. GFP and mCherry signals were thresholded and punctae were segmented for each image in a confocal stack, in which punctae were defined as containing at least 100 pixels, corresponding to 32.5 μm^2^. To determine whether GFP punctae contained mCherry signal, regions of a confocal stack that corresponded to GFP punctae, were measured in the mCherry channel. Similarly, to determine whether mCherry punctae contained GFP signal, regions of a confocal stack that corresponded to mCherry punctae were measured in the GFP channel.

### 2.6. Statistical Analysis

All statistical analyses were performed using SigmaStat 3.2 and graphs were generated using Graph Pad Prism 5.03 (Graph Pad Software; San Diego, CA, USA). A 1-way or 2-way ANOVA, or a Mann-Whitney Rank Sum test was used for analysis when appropriate. Values are presented as mean ± S.E.M and differences deemed significant when *p* ≤ 0.05.

## 3. Results

The transcriptional activity of many molluscan RXRs has been examined using mammalian cell lines, which may influence the availability or binding of coactivators [27]. Here, we developed a live CNS organ culture from *Drosophila* larvae in order to examine the ligand-binding and transcriptional activity of a molluscan RXR in the context of an invertebrate nervous system. To this end, we designed a ligand sensor system utilizing the RXR of the mollusc *Lymnaea stagnalis* (Figure 1A and Figure S1), which importantly, was capable of reporting both the expression, as well as the transcriptional activity of the sensor. 

The ligand sensor system consisted of 5 components: GFP, T2A, Neomycin, dT2A, and the LymRXR hinge and ligand-binding domain (LBD) fused to the GAL4 DNA-binding domain (DBD) containing a FLAG-tag (Figure 1A). This construct was stably integrated into the genome of *Drosophila melanogaster* to generate stable fly lines. The expression of this multi-component transgene was under the control of the HSP70 heat shock promoter, and upon heat shock, the genetic construct resulted in the expression of 3 distinct proteins: GFP, Neo and the LymRXR ligand sensor (Figure 1A). T2A sequences allowed for the translation of multiple independent proteins from a single RNA molecule. Expression of GFP and the LymRXR sensor as separate proteins from the same RNA molecule allowed us to positively identify cells expressing the LymRXR ligand sensor construct without the possibility of GFP-tagging impeding its function. 

Heat-shock was shown to successfully induce the expression of GFP (Figure 1B; mostly cytoplasmic as shown in the middle panel) in 3rd instar larval CNS hemispheres (fly line #2). When the LymRXR ligand sensor flies (fly line #2) were crossed with flies containing mCherry-NLS under the control of UAS (fly line #1), transcriptional activation by the LymRXR sensor was reported by expression of nuclear mCherry (Figure 1A). This genotype (containing both the LymRXR sensor and the UAS-mCherry-NLS reporter; fly line #3) will be hereafter referred to as the “LymRXR ligand sensor”. Expression of the LymRXR ligand sensor was thus reported by expression of GFP and transcriptional activity induced by the ligand sensor was reported by expression and targeted nuclear localization of mCherry. 

### 3.1. The LymRXR Ligand Sensor Responds to All-Trans RA in the Drosophila Larval CNS

Retinoid X receptors (RXRs) are known to bind the 9-*cis* RA isomer with high affinity in species of many different lineages, including vertebrates, annelids and molluscs. However, their ability to bind all-*trans* RA (atRA) is less clear, and discrepancies have been found from binding and transactivation studies. It is mainly thought that vertebrate RXRs do not bind atRA, though some invertebrate RXRs have been shown to [28], suggesting differences in binding affinities between vertebrate and invertebrate RXRs. Though RXR is canonically a nuclear ligand-activated transcription factor, LymRXR is present only in the cytoplasmic and membrane compartments of adult *Lymnaea* neurons [23]. Despite evidence for its role in non-genomic signaling in adult neurons [23], we have not yet determined whether LymRXR is involved in transcriptional regulation in *Lymnaea*, and which retinoid isomers might bind and activate this receptor. 

**Figure 2:**
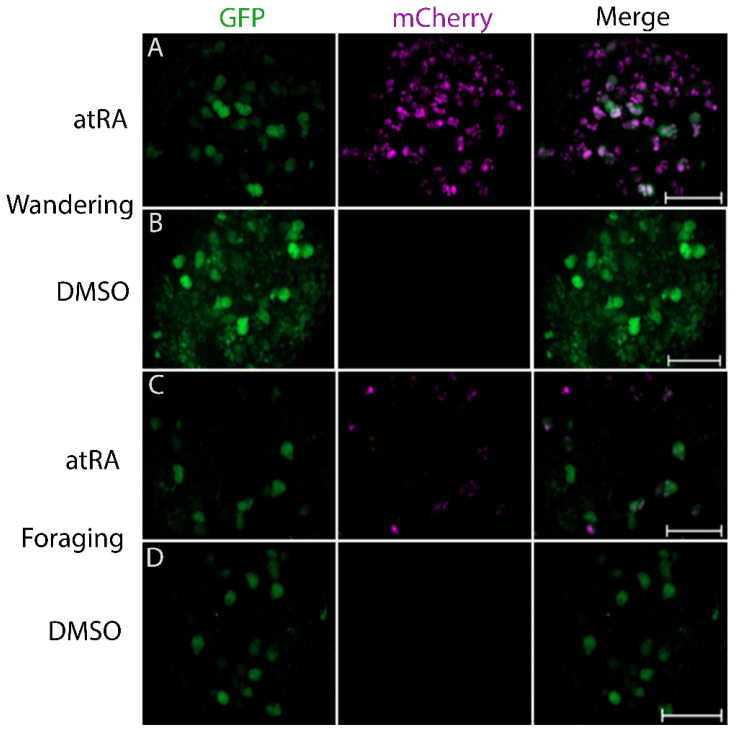
Activation of the LymRXR sensor by 10 µM all-*trans* RA. Representative examples of *Drosophila* CNS (individual hemispheres) exposed to either 0.1% DMSO (**B**,**D**) or 10 μM all-*trans* RA (atRA) (**A**,**C**) in either wandering (top panels) or foraging (lower panels) larvae following heat-shock. Images show sensor expression levels (GFP only), transcriptional activity of the sensor (mCherry only), and a merged representation. Though there are no significant differences in GFP levels, mCherry levels are clearly elevated following exposure to 10 μM atRA. Scale bars: 50 μm.

To determine whether the *Lymnaea* RXR activates gene transcription in response to atRA in a live invertebrate nervous system, we heat-shocked 3rd instar (foraging or wandering) LymRXR ligand sensor larvae. Larval CNS preparations were then incubated in either atRA (10 μM) or DMSO (0.1%) for 24 h, followed by live imaging. As protein expression mediated by heat-shock can be stochastic, cell specific, and can vary between individual animals and experimental conditions [29], we first examined levels of ligand sensor expression (GFP) between different larval developmental stages (foraging versus wandering) as well as treatment conditions (atRA versus DMSO) in response to heat-shock. Representative images of CNS hemispheres are shown in Figure 2.

**Figure 3:**
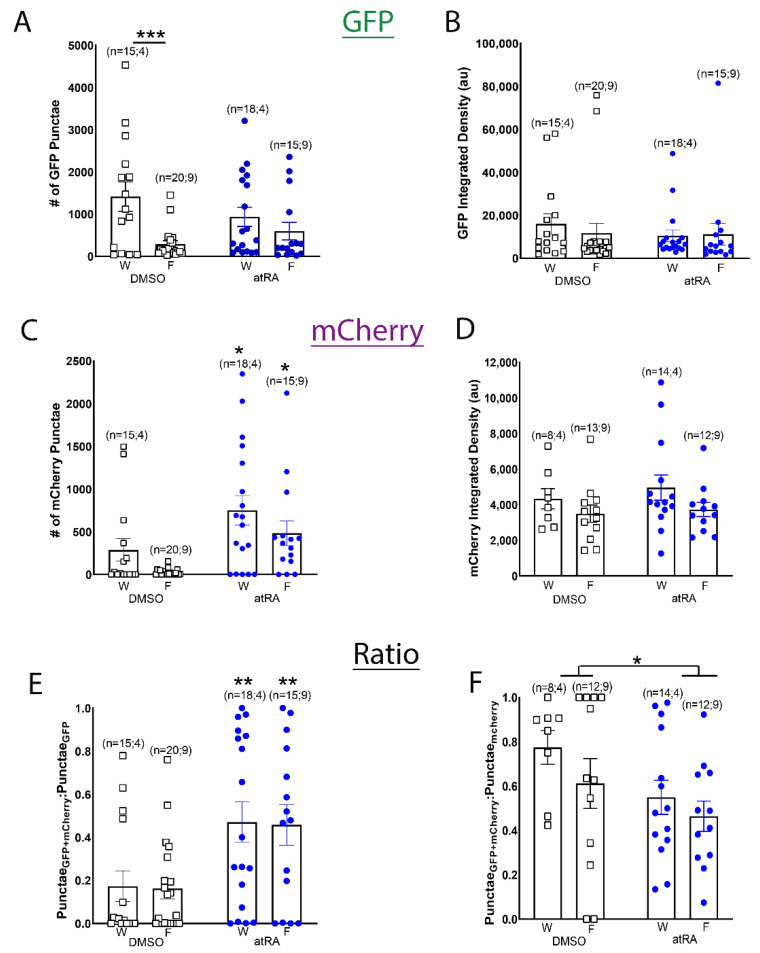
Quantification of GFP and mCherry expression following heat-shock and exposure of *Drosophila* CNS to either 10 μM all-*trans* RA or 0.1% DMSO. (**A**) There is no significant difference in the number of GFP punctae between all-*trans* RA (atRA) and DMSO treatments, though CNS incubated in 0.1% DMSO show a significant difference between wandering (W) and foraging (F) larvae. (**B**) There is no significant difference in the GFP integrated density, either between atRA and DMSO treatments, or between wandering and foraging larvae. (**C**,**D**) There is a significant increase in the number of mCherry punctae following atRA treatment compared to DMSO (**C**), though no significant difference in the integrated density (**D**). (**E**) The proportion of GFP punctae that contain mCherry is significantly increased following exposure to atRA, in both foraging and wandering larvae, compared to vehicle controls. These data demonstrate that atRA increases the number of cells in which the LymRXR sensor is transcriptionally active. (**F**) There is a significant difference in the proportion of mCherry punctae that contain GFP between DMSO and RA treatments. * *p* < 0.05; ** *p* < 0.01; *** *p* < 0.001. Numbers in brackets represent total number of CNS, followed by the number of separate trials (replicates).

As a proxy for heat-shock-induced ligand sensor expression, the number of GFP-positive punctae and the integrated density of GFP fluorescence were determined (Figure 3A,B). A two-way ANOVA of the number of GFP punctae (*F*_(1,64)_ = 0.147; *p* = 0.703; Figure 3A) and their integrated density (*F*_(1,64)_ = 0.469; *p* = 0.496; Figure 3B) revealed no significant differences between treatment conditions (DMSO and atRA) as expected, though a significant difference in ligand sensor expression between the different developmental stages (wandering versus foraging larvae) occurred. In DMSO-treated CNS from wandering 3rd instar larvae, there was a significant increase in the number of GFP punctae (*F*_(1,64)_ = 10.941; *p* = 0.002), compared to their foraging stage counterparts (Figure 3A). However, larval developmental stage had no effect on GFP punctae integrated density (F_(1,64)_ = 0.168; *p* = 0.683; Figure 3B). These data suggest that heat shock-induced expression of the LymRXR sensor can vary between larval developmental stages, regardless of experimental treatments.

To determine whether atRA treatment impacts transcription mediated by the LymRXR ligand sensor, we quantified mCherry expression. The number of mCherry punctae serves as a measure of the number of cells in which the ligand sensor has been activated, whereas the mCherry integrated density indicates the degree of ligand sensor activity in those cells (corresponding to the extent of gene transcription). Representative images in Figure 2 illustrate that 10 μM atRA increased the mCherry expression in larval CNS hemispheres; a two-way ANOVA of the number of mCherry punctae revealed a significant effect of treatment (*F*_(1,64)_ = 13.158; *p* < 0.001; Figure 3C), with an atRA-induced increase in the number of mCherry punctae in the CNS of both wandering and foraging larvae, compared to vehicle controls. These data indicate that atRA increased the number of cells in which the LymRXR sensor induced transcriptional activation. There was also a significant effect of developmental stage on ligand sensor activity (*F*_(1,64)_ = 4.324; *p* = 0.042), with wandering larvae expressing more mCherry punctae than their isogenic foraging counterparts. In contrast, a two-way ANOVA of mCherry integrated density revealed no significant effect of treatment (*F*_(1,42)_ = 0.533; *p* = 0.469; Figure 3D), indicating that atRA did not appear to increase the quantity of mCherry protein produced by activation of the LymRXR sensor. 

As a more stringent quantitative measure of ligand sensor activation, and to account for differences in CNS size, sensor expression and variation in the number of cells competent to respond to atRA, we measured the proportion of GFP punctae that contained mCherry (Figure 3E). A two-way ANOVA revealed a significant effect of treatment (*F*_(1,64)_ = 14.518; *p* < 0.001); atRA significantly increased the proportion of GFP punctae that contained mCherry in both foraging and wandering larvae compared to vehicle controls, indicating that atRA increased the number of cells in which the LymRXR sensor was transcriptionally active, and thus activated the LymRXR sensor. 

To determine whether cells in which the LymRXR sensor was transcriptionally active also contained measurable ligand sensor expression, we examined the proportion of mCherry containing nuclei that also contained GFP. Most CNS preparations treated with either atRA or DMSO exhibited a GFP:mCherry ratio of less than 1 (Figure 3F), indicating that many cells had sensor expression (GFP) below the threshold of detection, despite the presence of mCherry. It should be noted that UAS-mCherry-NLS alone (fly line #1) did not exhibit non-specific ‘leaky’ reporter expression (data not shown). A two-way ANOVA revealed a significant effect of treatment (*F*_(1,42)_ = 4.356; *p* = 0.043), but not of larval developmental stage. The proportion of mCherry punctae that contained GFP was significantly decreased in atRA compared to DMSO, suggesting that cells in which sensor expression (GFP) was below the threshold of detection, were more responsive in the presence of atRA than in the presence of DMSO. Thus, although our assessment of the proportion of GFP punctae that contained mCherry was more stringent, it was likely an underestimation of the number of cells competent to respond to atRA. We limit all further analysis in this manuscript to the proportion of GFP punctae that contained mCherry in CNS from only foraging 3rd instar larvae following heat-shock (unless otherwise stated). 

The concentration of atRA in the *Lymnaea* CNS was previously estimated to be approximately 0.7 μM [30]. We have routinely used low micromolar concentrations of atRA when examining the physiological roles of retinoids in neuronal signaling, so we next sought to determine whether the lower concentrations of 5 or 1 μM atRA would also activate the ligand sensor to induce transcriptional regulation. We heat-shocked LymRXR sensor larvae and incubated the CNS for 24 h in either 5 or 1 μM atRA (or the equivalent concentration of DMSO), and again quantified reporter activity in CNS hemispheres by live fluorescence imaging. 

**Figure 4:**
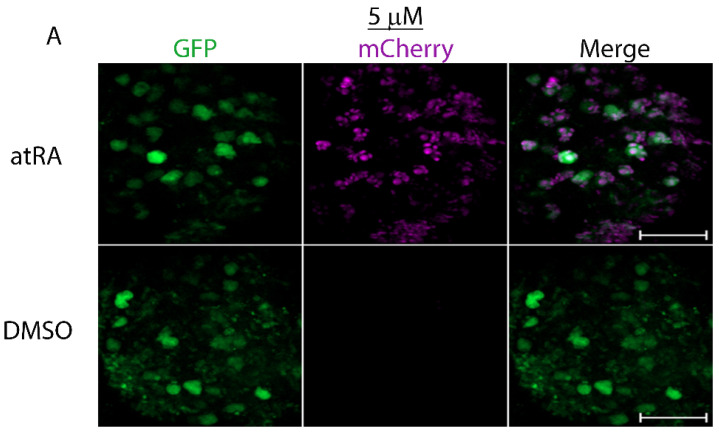

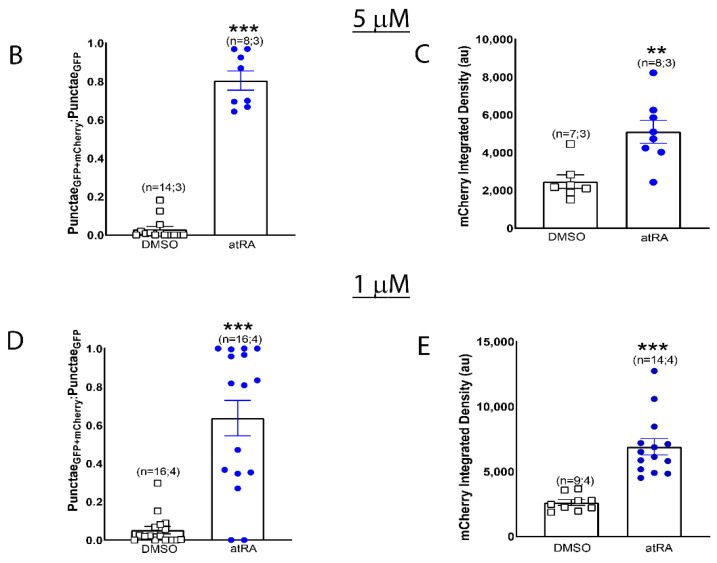
Lower concentrations of both 5 µM and 1 µM all-*trans* RA significantly enhance transcriptional activity of the sensor. (**A**) Representative images of sensor activation and transcriptional activity in CNS hemispheres by 5 µM all-*trans* RA (atRA), but not by 0.05% DMSO, following heat-shock. (**B**,**D**) Quantitative analysis indicates a significantly higher proportion of GFP punctae also containing mCherry following exposure to both 5 µM atRA (**B**) and 1 µM atRA (**D**) and a significantly higher mCherry integrated density following exposure to both 5 µM atRA (**C**) and 1 µM atRA (**E**). ** *p* < 0.01; *** *p* < 0.001, compared to DMSO. Numbers in brackets represent the total number of CNS and number of separate trials. Scale bars: 50 μm.

Representative images in Figure 4 illustrate increased mCherry expression throughout a CNS hemisphere following exposure to 5 μM atRA. Both 5 μM (Mann-Whitney Rank Sum test; *p <* 0.001; Figure 4B) and 1 μM (Mann-Whitney Rank Sum test; *p <* 0.001; Figure 4D) significantly increased the proportion of GFP punctae that contained mCherry, compared to DMSO. In addition, both 5 μM (Mann-Whitney Rank Sum test; *p <* 0.01; Figure 4C) and 1 μM (Mann-Whitney Rank Sum test; *p <* 0.001; Figure 4E) significantly increased mCherry integrated density. These data suggest that lower micromolar concentrations of atRA increased the proportion of cells in which the LymRXR sensor was transcriptionally active, but also appeared to increase the degree of ligand sensor activity. 

### 3.2. Both All-Trans and 9-Cis Retinoid Isomers Activate LymRXR to a Similar Extent

Vertebrate RXRs are generally reported to preferentially bind 9-*cis* RA, whereas some invertebrate RXRs have been reported to bind both all-*trans* and 9-*cis* RA isomers with equal affinity [28]. Although both all-*trans* and 9-*cis* isomers of retinoic acid were previously found in the CNS (and hemolymph) of *Lymnaea*, we have previously described differing physiological effects of these two isomers on adult *Lymnaea* neurons [31]. We next sought to determine whether all-*trans* or 9-*cis* RA differentially influence transcriptional regulation by LymRXR. To do so, we heat-shocked LymRXR sensor larvae and incubated their CNS for 24 h in either 1 μM atRA, 1 μM 9-*cis* RA or DMSO (0.01%) and again quantified reporter activity by live fluorescence imaging.

**Figure 5:**
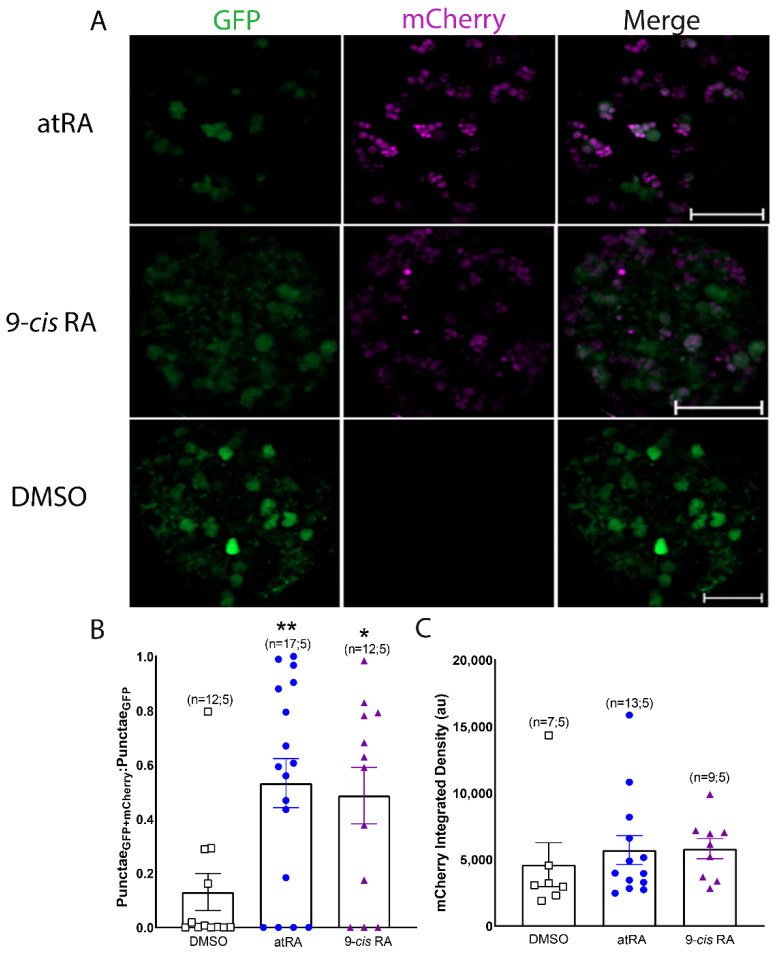
All-*trans* and 9-*cis* RA isomers show equivalent activation of the LymRXR sensor. (**A**) Representative images of CNS hemispheres following heat-shock, showing sensor expression (GFP) and transcriptional activity (mCherry) after exposure to either 1 µM atRA or 1 µM 9-*cis* RA, but no transcriptional activity following exposure to 0.01% DMSO, despite sensor expression (GFP only). (**B**,**C**) Quantitative analysis indicates a significantly higher proportion of GFP punctae containing mCherry following exposure to either 1 µM atRA or 1 µM 9-*cis* RA, compared to DMSO controls (**B**), though there is no significant difference in mCherry integrated density (**C**). * *p* < 0.05; ** *p* < 0.01, compared to DMSO. Numbers in brackets represent the total number of CNS and the number of separate trials. Scale bars: 50 μm.

Representative images in Figure 5A demonstrate that both all-*trans* and 9-*cis* RA increased mCherry expression in larval hemispheres. A one-way ANOVA of the proportion of GFP punctae that contained mCherry revealed a significant difference across conditions (*F*_(2,38)_ = 5.741; *p* = 0.008), with a significant increase following treatment with either atRA or *9-cis* RA, compared to DMSO (Figure 5B). Importantly, no significant difference was found between all-*trans* and *9-cis* RA isomers (*p* *=* 1.0), suggesting that both isomers increased (to a similar extent) the proportion of cells in which the LymRXR sensor was transcriptionally active. A one-way ANOVA on ranks of mCherry integrated density revealed no significant difference between groups (*p* *=* 0.166; Figure 5C), suggesting that the degree of transcriptional activity of the LymRXR sensor was not significantly different between treatments with either of the retinoid isomers.

### 3.3. Sensor Activation by a Vertebrate RXR Agonist

Our results indicate that, in contrast to previous reports of vertebrate RXR ligand selectivity, LymRXR appears to be equally activated by both all-*trans* and 9-*cis* RA. To further probe the binding properties of the LymRXR, we next compared the activity of various known vertebrate ligands, including the vertebrate RXR-selective agonist SR11237 (1 μM), and the vertebrate RAR-selective synthetic retinoid EC23 (1 μM). Both of these ligands were previously shown to exert physiological effects in *Lymnaea* neurons [15] and to affect *Lymnaea* behavior [14,32] at these concentrations. 

**Figure 6:**
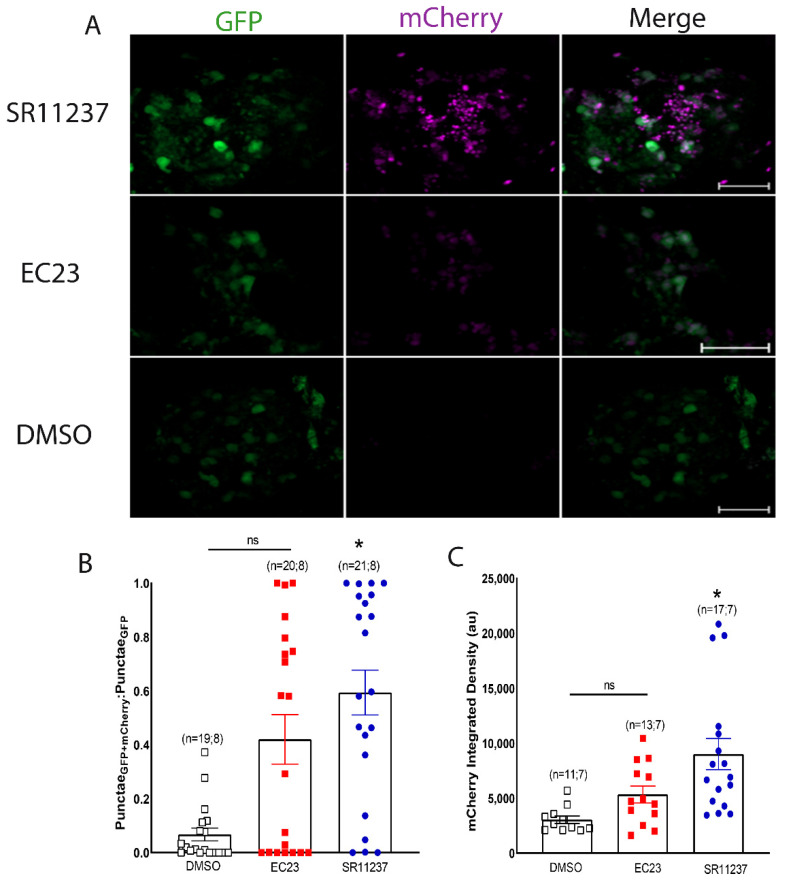
Sensor activation by a vertebrate RXR agonist. (**A**) Representative images of CNS hemispheres following heat-shock, showing the highest level of sensor activation by the vertebrate RXR agonist SR11237 (1 µM), though also some activation by the synthetic retinoid and RAR-selective agonist, EC23 (1 µM). (**B**,**C**) Despite no significant increase in the proportion of GFP punctae also containing mCherry in the CNS exposed to EC23, compared to vehicle controls (0.01% DMSO), there is some level of transcriptional activity by the sensor in response to EC23. The proportion of GFP punctae also expressing mCherry is, however, significantly higher in CNS exposed to the vertebrate RXR agonist SR11237 (**B**), as is the mCherry integrated density, compared to DMSO controls (**C**). * *p* < 0.05, compared to DMSO. Numbers in brackets represent total number of CNS and number of separate trials. Scale bars: 50 μm.

We heat-shocked LymRXR sensor larvae and incubated their CNS for 24 h in either 1 μM SR11237, 1 μM EC23, or DMSO (0.01%) and again quantified reporter activity by live fluorescence imaging, with representative images shown in Figure 6A. A one-way ANOVA on ranks (H = 14.601; *p* = 0.0014) of the proportion of GFP punctae that contained mCherry revealed a significant effect of agonist treatment (Figure 6B). However, post-hoc analysis revealed that only SR11237 significantly increased the proportion of GFP punctae that contained nuclear mCherry, compared to DMSO. Similarly, a one-way ANOVA on ranks (H = 15.181; *p* < 0.001) determined that SR11237, but not EC23, significantly increased the mCherry integrated density, compared to DMSO (Figure 6C). It should be noted that although EC23 did not exhibit any significant increase compared to DMSO, there was also no significant difference between the effects of EC23 and those of SR11237, indicating some level of transcriptional activation by EC23. Collectively, these data suggest that the LymRXR has conserved ligand-binding properties as only the vertebrate RXR-selective agonist significantly activated the LymRXR, compared to DMSO. 

### 3.4. Evidence for Endogenous Retinoid Activity in the Developing CNS of Drosophila Larvae

In vertebrates, Vitamin A deficiency results in abnormal patterning and development of the nervous system, due to reduced RAR and RXR signaling. Vitamin A deficiency, however, does not result in gross developmental abnormalities in *Drosophila*, a species that does not possess either an RAR or a canonical RXR; that is, the RXR homologue in *Drosophila* (Ultraspiracle [USP]), does not bind retinoids. As such it has been suggested that *Drosophila* primarily utilizes retinoids, particularly 11-*cis* retinaldehyde, for rhodopsin generation. However, RA can also affect cellular activity by binding to noncanonical nuclear receptors, as well as kinases. 

**Figure 7:**
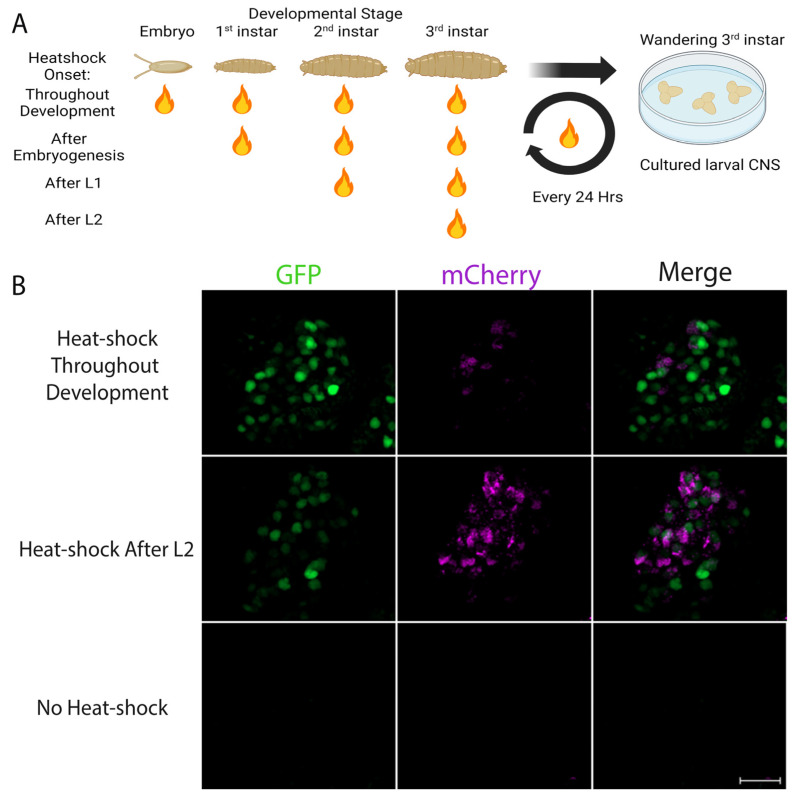

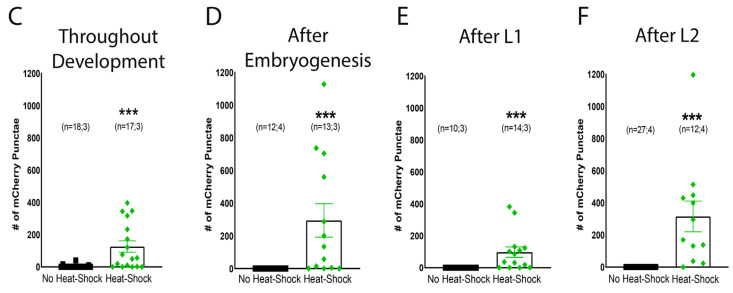
Activation of the sensor by endogenous molecules during larval development. (**A**) Schematic showing the heat-shock paradigm implemented during different stages of *Drosophila* development (image made with Biorender). (**B**) Repetitive heat-shock throughout *Drosophila* larval development (upper panels), or initiated after L2 (middle panels), induces sensor expression (GFP) in larval hemispheres, but not in the absence of heat-shock (lower panels). Despite the absence of any exposure to exogenous retinoids, significant transcriptional activation (mCherry expression) is observed in larval CNS hemispheres, after heat-shock throughout development and when heat-shocked after L2. (**C**–**F**) Quantification of the number of mCherry punctae indicates significant sensor activation occurred when heat-shocked throughout development (**C**), after embryogenesis (**D**), after L1 (**E**) or after L2 (**F**), compared to no heat-shock controls. Numbers in brackets represent total number of CNS and the number of separate trials. *** *p* ≤ 0.001 compared to no heat-shock controls. Scale bar: 50 μm.

To test whether the LymRXR sensor could detect retinoic acid-like molecules in vivo during *Drosophila* development, LymRXR sensor progeny were heat-shocked every 24 h, beginning at specific developmental stages. In particular, heat-shock treatment was either applied throughout development, or was initiated following embryogenesis (1st instar), following L1 (2nd instar), or following L2 (3rd instar) (Figure 7A). Once these larvae, heat-shocked at different developmental stages, reached the wandering 3rd instar larval stage, their CNS were harvested and cultured for live imaging 24 h later. As a control, we performed live imaging on CNS preparations from larvae that were not subjected to heat- shock during development, and as expected, these exhibited little to no GFP expression. 

Detection of sensor activity was quantified using the number of mCherry punctae (rather than the proportion of GFP punctae that contained mCherry), to allow comparison with their non-heat-shocked control counterparts. Representative images in Figure 7B illustrate that ligand sensor activation occurred when larvae were heat-shocked throughout development and also when heat-shocked after L2, though there was no mCherry expression in control larvae that were not heat-shocked. A Mann-Whitney rank sum test revealed a significant increase in the number of mCherry punctae following heat-shock at each developmental stage (*p* ≤ 0.001), compared to non-heat-shocked controls (Figure 7C–F). 

In order to rule out that prolonged heat-shock paradigms may have caused accumulation of sensor expression over time, GFP expression following heat-shock at different developmental stages was analyzed. A one-way ANOVA on ranks of the number of GFP punctae (H = 39.64; *p* < 0.001) and the GFP integrated density (H = 29.50; *p* < 0.001) revealed no significant differences in sensor expression (GFP) between CNS preparations that were heat-shocked at different stages of development (only differences compared to non-heat-shocked controls; Figure S2). 

Overall, these data suggest that there may indeed be nuclear signaling and transcriptional regulation mediated by retinoic acid or retinoid-like molecules during CNS development in *Drosophila* larvae. Furthermore, as these data indicate that LymRXR sensor expression and activation occurred to a similar extent following heat-shock across any of the larval stages, endogenous retinoid activity might be occurring primarily during the 3rd instar larvae stage.

## 4. Discussion

In this study, we generated a novel LymRXR ligand sensor fly line that reported both the expression and the transcriptional activity of the ligand sensor. This LymRXR ligand sensor system activated gene transcription in the presence of either 9-*cis* RA or all-*trans* RA as well as various synthetic retinoid agonists. It was also able to report the presence of possible endogenous retinoid signaling during *Drosophila* embryonic and/or larval development. Thus, in addition to providing insights into transcriptional regulation by a molluscan RXR, this ligand sensor also provides a genetic tool to examine potential retinoid-based signaling in *Drosophila*. 

### 4.1. RXR and Retinoids in Lymnaea

We have previously shown that the *Lymnaea* CNS (and hemolymph) contains retinoic acid [30] and have provided evidence that LymRXR plays a role in regulating embryonic development and axon guidance [23]. Retinoic acid is a well-known developmental morphogen [33] and in *Lymnaea* embryos, RXR protein is present in early trochophores (36 to 60 h of development). Both 9-*cis* RA (0.1 µM) and a vertebrate RXR agonist (PA024) caused defects in eye and shell development in *Lymnaea* embryos [23], supporting a role for retinoids [34] and RXR [23] in molluscan development. Though *Lym*RXR was found in the nucleus during embryonic development [23], its role in transcriptional regulation in response to various retinoids has not been studied until now.

In contrast to its nuclear localization in embryos, western blotting and immunostaining of adult CNS failed to detect *Lym*RXR in the nucleus, though it was detected in the cytoplasm and membrane, and was localized to axonal tracts of the neuropil [23]. Indeed, in adult neurons, functional studies have implicated *Lym*RXR in non-genomic signaling; growth cone turning mediated by both RA and vertebrate RXR agonists occurs in isolated, transected neurites (in the absence of the cell body and nucleus). Despite our inability to detect nuclear *Lym*RXR in the adult CNS, we now provide evidence it may be capable of activating gene transcription in response to retinoic acid (though we cannot discount the possibility that the Gal4 fusion protein has differential transcriptional activity in comparison to the full-length endogenous receptor). 

These findings raise the question of whether LymRXR signaling might have a genomic function during embryonic development, but later becomes predominantly non-genomic, at least in the adult nervous system. Interestingly, however, we have also shown that LymRXR (as with vertebrate RXR) is involved in long-term memory formation, a process that generally requires gene transcription. This suggests that *Lym*RXR signaling might mediate both genomic and non-genomic signaling in the adult molluscan nervous system. Indeed, vertebrate RXRs contribute to working memory [35] and activate neuronal gene programs to regulate dendritic complexity [36]. However, vertebrate RXRs are also involved in mGluR-mediated long-term depression [37], a form of synaptic depression that typically involves local protein synthesis at synapses, suggesting that vertebrate RXRs might also affect cellular and synaptic function via non-genomic mechanisms.

### 4.2. Molluscan RXRs and Retinoid Binding

The RXR from the mollusc *B. glabrata* was the first molluscan RXR shown to functionally interact with vertebrate RXR ligands [22]. Its LBD shares 81% identity with mouse RXRα; indeed, many molluscan RXR LBDs, including that of the LymRXR, show greater identity to vertebrate RXRs than to *Drosophila* USP (RXR ortholog). BgRXR was examined in mammalian cells lines and shown to transactivate transcription of a reporter gene in the presence of 9-*cis* RA [22]. However, this transactivation by BgRXR in the mammalian cell lines was less than for mouse RXR, and the authors suggested that either 9-*cis* RA was not the optimal ligand for BgRXR, or that there was possibly suboptimal interaction between the molluscan receptor and available endogenous transcriptional co-activators. 

The transcriptional activities of three different gastropod RXRs: *T*. *clavigera*, *N. Lapillus* and *B. japonica*, were subsequently examined using COS-1 cells in vitro [10] and all were significantly induced by 1 µM 9-*cis* RA, as well as the vertebrate RXR-selective agonist, PA024. Jellyfish RXR also binds 9-*cis* RA [1], as does the RXR from the simple multicellular organism, *Trichoplax adhaerens* [38], supporting the conclusion that ancestral RXRs bind retinoids. A recently cloned RXR from the Pacific oyster (*C. gigas*), also transcriptionally activated by 9-*cis* RA, was able to form a heterodimer with the *Cg*TR, providing evidence that molluscan RXRs also heterodimerize with other members of the nuclear receptor superfamily [39]. 

It is not surprising that invertebrate RXRs are activated by 9-*cis* RA (first identified as a high affinity ligand for vertebrate RXRs). The amino acids lining the ligand binding pocket, known to interact with retinoids in vertebrate RXRα, are conserved in molluscan RXRs [22,23]. In light of these conserved residues, activation of RXRs by the all-*trans* RA isomer has been reported in some molluscan species [40], which is perhaps unexpected, considering vertebrate RXRs exhibit little, to no binding of all-*trans* RA [4]. These data thus provide support for overlapping, but differing, ligand binding properties of molluscan and vertebrate RXRs. It has been suggested that ligand-receptor pairs might indeed exhibit changes in their binding specificities or activation profiles, and strict conservation of residues lining the LBD pocket has been deemed insufficient to guarantee ligand-receptor interaction [6]. 

Although 9-*cis* RA is a high affinity ligand for vertebrate RXRs, vertebrate nervous tissues are thought to have extremely low levels of 9-*cis* RA, raising concerns about its validity as an endogenous RXR ligand. Recently, however, 9-*cis*-13,14-dihydroretinoic acid was identified as a RXR ligand with higher endogenous tissue levels in mice, indicating it may be the elusive natural ligand for vertebrate RXRs [5]. In contrast to vertebrates, the *Lymnaea* CNS contains high levels of both 9-*cis* and all-*trans* RA, and as such, RXR signaling might be activated by both RA isomers in vivo. Indeed, we provide evidence here that both 9-*cis* and all-*trans* RA isomers bind to the *Lym*RXR and appear to activate our ligand sensor system to a similar extent. 

It has been proposed that all-*trans* RA can spontaneously isomerize to 9-*cis* RA [41], and though we cannot rule out the possibility that such isomerization might have occurred within the *Drosophila* CNS, it unlikely accounts for our results. Other studies indicate that there is no evidence for isomerization of *all-trans* RA to *9-cis* RA [28,42,43], and it has also been proposed that 9-*cis* RA might be synthesized through a different pathway from *all-trans* RA [44], suggesting distinct signaling of these two isomers. Indeed, we have previously shown selective and differential effects of 9-*cis* and all-*trans* RA in physiological assays [31]. Interestingly, in addition to activation of our ligand sensor by all-*trans* RA, we also showed that a synthetic retinoid (EC23), considered a selective RAR agonist [45,46] also activated LymRXR (though to a lesser extent than the vertebrate RXR ligand SR11237). These data further support differing binding properties between vertebrate and molluscan RXRs, as EC23 does not appear to bind to or activate vertebrate RXRs [45].

RXR receptor organization may also differ between vertebrates and invertebrates. In the absence of ligand, vertebrates RXRs form tetramers, which upon ligand binding, dissociate into homodimers that then mediate gene transcription. In contrast, molluscan RXR receptors may mediate gene transcription as tetramers. For example, the crystal structure of *B. glabrata* RXR revealed that it is capable of binding retinoic acid and coactivators as a tetramer [47], potentially resulting in differential transcriptional regulation. 

### 4.3. Retinoid Signaling in Drosophila

*RXR* is considered an ancient gene but has been subjected to a number of functional shifts through evolution. For example, the RXR homologue (USP) in *Drosophila* does not respond to 9-*cis* RA [48]. Indeed, the ability to bind 9-*cis* RA has been lost in many arthropods, (although the locust RXR/USP has been shown to bind both all-*trans* and 9-*cis* RA [28]). Vertebrate-like RXRs are thus absent from *Drosophila* [48], providing an ideal live invertebrate CNS system to examine transcriptional regulation by a molluscan RXR.

Although *Drosophila* does not contain a retinoid-binding RXR (or RAR) ortholog, it does contain transporters and synthetic enzymes involved in retinoid metabolism. We found that expression of our LymRXR ligand sensor during *Drosophila* larval development, resulted in activation of the ligand sensor in the absence of exogenously applied retinoids, likely occurring primarily during the 3rd instar larval stage. This could have resulted from binding of endogenous RA (or RA-like molecules) to the LymRXR ligand sensor. 

We cannot rule out the possibility that endogenous activation of our LymRXR ligand sensor during *Drosophila* development, occurred as a result of heterodimerization with an activated nuclear receptor. The human RXR can heterodimerize with *Drosophila* nuclear receptors, one example being the ecdysone receptor which is involved in the developmental transition between the wandering 3rd instar larval stage and pupation, in response to ecdysone [49]. However, expression of a human RXR ligand sensor during larval development when ecdysone signaling occurs, exhibited no activation [48]. In addition to retinoic acid, vertebrate RXRs are also activated by docosahexaenoic acid (DHA), although it is highly unlikely that DHA was responsible for activation of our ligand sensor system, as *Drosophila* does not produce DHA [50]. Juvenile hormone (JH) is a developmental molecule found in *Drosophila* that has a similar structure to retinoic acid [51], though does not activate human RXR receptors, and rather inhibits activation of human RXR ligand sensors in *Drosophila* larval brain cultures [48]. It is thus also considered unlikely that JH might be activating the LymRXR ligand sensor. 

Assuming that RA, or RA-like molecules, are responsible for activation of the LymRXR ligand sensor during normal *Drosophila* larval development, what is the source of these RA-like molecules, what roles do they play and what might their target(s) be? When we examined the effects of exogenously applied retinoids, the CNS were removed from the animal immediately following heat-shock, and our results indicated that the vehicle controls exhibited no (or minimal) sensor activation. However, when larvae were heat-shocked throughout various developmental stages (including 3rd instar larvae), the CNS were not immediately removed from the larvae and significant sensor activation occurred (in the absence of exogenous retinoids). These data suggest that the endogenous retinoid-like molecules might originate from outside the fly CNS. 

RA is synthesized through oxidation of retinaldehyde by RALDH, an aldehyde dehydrogenase (ALDH) family member. Disruption of ALDH in *Drosophila* prevents both the developmental delay thought to be required for regeneration (following damage to imaginal tissues) [52], as well as induces spontaneous degeneration of axons in *Drosophila* CNS [53]. These findings provide support that retinoid signaling might be involved in *Drosophila* development and/or maintenance of the nervous system. Vitamin A-deficient diets do not, however, produce gross developmental abnormalities in *Drosophila*, and as such, it is thought that retinoid signaling plays a role only in chromophore development. 

Although *Drosophila* lack a RAR or an RXR/USP that bind retinoids, RA can bind to other nuclear receptors, such as peroxisome proliferator activated receptor (PPAR), and so might activate other nuclear receptors in *Drosophila*. RA can also bind directly to protein kinase C [54] which is ubiquitously important for signaling in the nervous system and may be a pathway by which retinoids exert effects in *Drosophila*. It is thus possible that retinoid signaling does play a role in the development of *Drosophila*, independently of RXR (and RAR) signaling. If so, the absence of gross abnormalities following Vitamin A deficiencies might be due either to a redundancy in signaling pathways, or that deficiencies may exert a far more subtle effect. For example, there might be adverse effects of defective RA signaling on either learning and memory, or locomotion, neither of which have yet been tested. The possibility of a role of retinoid signaling in *Drosophila* nervous system development and function thus requires further investigation. 

In summary, our novel ligand sensor system can be used to quantitatively map the spatio-temporal distribution of retinoid signaling during *Drosophila* development in vivo, highlighting the utility and versatility of this sensor system. Importantly, for our studies of retinoid function in the molluscan CNS, we were now able to demonstrate that both endogenous retinoid isomers could induce transcriptional activation through the LymRXR. Despite our previous findings that the LymRXR exhibited non-nuclear localization in the adult CNS, our data now support the possibility that the RXR may indeed mediate both non-genomic and genomic functions in the *Lymnaea* nervous system. 

## Supplementary Materials

The following supporting information can be downloaded at: https://www.mdpi.com/article/10.3390/cells11162493/s1, Figure S1: The DNA sequence of the LymRXR sensor construct; Figure S2: Quantification of sensor expression following different heat-shock paradigms.
