# Supplementary material for: Molluscan RXR Transcriptional Regulation by Retinoids in a Drosophila CNS Organ Culture System
Source: Cells. 2022 Aug 11;11(16):2493. doi: 10.3390/cells11162493 (PMC9406730; doi:10.3390/cells11162493)
Supplement: Supplementary file 1 [file cells-11-02493-s001.zip › cells-1703815-supplementary.pdf]

Supplementary Materials:

ATGGTGAGCAAGGGCGAGGAGCTGTTCACCGGGGTGGTGCCCATCCTGGTCGAGCTGGA
CGGCGACGTAAACGGCCACAAGTTCAGCGTGTCCGGCGAGGGCGAGGGCGATGCCACCT
ACGGCAAGCTGACCCTGAAGTTCATCTGCACCACCGGCAAGCTGCCCGTGCCCTGGCCCA
CCCTCGTGACCACCCTGACCTACGGCGTGCAGTGCTTCAGCCGCTACCCCGACCACATGA
AGCAGCACGACTTCTTCAAGTCCGCCATGCCCGAAGGCTACGTCCAGGAGCGCACCATCT
TCTTCAAGGGCGACGGCAACTACAAGACCCGCGCCGAGGTGAAGTTCGAGGGCGACACC
CTGGTGAACCGCATCGAGCTGAAGGGCATCGACTTCAAGGAGGACGGCAACATCCTGGG
GCACAAGCTGGAGTACAACTACAACAGCCACAACGTCTATATCATGGCCGACAAGCAGA
AGAACGGCATCAAGGTGAACTTCAAGATCCGCCACAACATCGAGGACGGCAGCGTGCAG
CTCGCCGACCACTACCAGCAGAACACCCCCATCGGCGACGGCCCCGTGCTGCTGCCCGA
CAACCACTACCTGAGCACCCAGTCCGCCCTGAGCAAAGACCCCAACGAGAAGCGCGATC
ACATGGTCCTGCTGGAGTTCGTGACCGCCGCCGGGATCACTCTCGGCATGGACGAGCTGT
ACAAGGAGGGCAGAGGAAGTCTTCTAACATGCGGTGACGTGGAGGAGAATCCCGGCCCT
GCTAGCATGGGATCGGCCATTGAACAAGATGGATTGCACGCAGGTTCTCCGGCCGCTTGG
GTGGAGAGGCTATTCGGCTATGACTGGGCACAACAGACAATCGGCTGCTCTGATGCCGCC
GTGTTCCGGCTGTCAGCGCAGGGGCGCCCGGTTCTTTTTGTCAAGACCGACCTGTCCGGTG
CCCTGAATGAACTGCAAGACGAGGCAGCGCGGCTATCGTGGCTGGCCACGACGGGCGTT
CCTTGCGCAGCTGTGCTCGACGTTGTCACTGAAGCGGGAAGGGACTGGCTGCTATTGGGC
GAAGTGCCGGGGCAGGATCTCCTGTCATCTCACCTTGCTCCTGCCGAGAAAGTATCCATC
ATGGCTGATGCAATGCGGCGGCTGCATACGCTTGATCCGGCTACCTGCCCATTCGACCAC
CAAGCGAAACATCGCATCGAGCGAGCACGTACTCGGATGGAAGCCGGTCTTGTCGATCA
GGATGATCTGGACGAAGAACATCAGGGGCTCGCGCCAGCCGAACTGTTCGCCAGGCTCA
AGGCGAGCATGCCCGACGGCGAGGATCTCGTCGTGACCCATGGCGATGCCTGCTTGCCGA
ATATCATGGTGGAAAATGGCCGCTTTTCTGGATTCATCGACTGTGGCCGGCTGGGTGTGGC
GGACCGCTATCAGGACATAGCGTTGGCTACCCGTGATATTGCTGAAGAACTTGGCGGCGA
ATGGGCTGACCGCTTCCTCGTGCTTTACGGTATCGCCGCTCCCGATTCGCAGCGCATCGCC
TTCTATCGCCTTCTTGACGAGTTCTTCGAAGGACGCGGCAGCCTACTGACTTGCGGAGATG
TCGAAGAGAACCCTGGCCCTATGGACTACAAAGACCATGACGGCGATTATAAAGATCAT
GACATCGACTACAAGGATGACGATGACAAGGAGAACCTGTACTTCCAGTCCGATATCAA
GCTACTGTCTTCTATCGAACAAGCATGCGATATTTGCCGACTTAAAAAGCTCAAGTGCTCC
AAAGAAAAACCGAAGTGCGCCAAGTGTCTGAAGAACAACTGGGAGTGTCGCTACTCTCC
CAAAACCAAAAGGTCTCCGCTGACTAGGGCACATCTGACAGAAGTGGAATCAAGGCTAG
AAAGACTGGAACAGCTATTTCTACTGATTTTTCCTCGAGAAGACCTTGACATGATTTTGAA
AATGGATTCTTTACAGGATATAAAAGCATTGTTAACAGGATTATTTGTACAAGATAATGT
GAATAAAGATGCCGTCACAGATAGATTGGCTTCAGTGGAGACTGATATGCCTCTAACATT
GAGACAGCATAGAATAAGTGCGACATCATCAGCTAGCCAGAGAGTGAAGGAAAAAGGG
GATGGGGAGGTAGAGAGCACCAGTGGAGCCAACAATGACATGCCAGTAGAACAGATCTT
GGAAGCTGAGCTGGCTGTTGACCCTAAGATAGACACATACATAGATGCACAGAAAGATC
CAGTGACCAACATATGTCAGGCAGCAGATAAACAGCTCTTCACTTTGGTAGAGTGGGCCA
AACGAATACCTCATTTCACAGAGCTGCCCTTGGAGGATCAAGTTATTCTATTACGAGCAG
GTTGGAATGAGCTCCTCATAGCAGGCTTCTCCCACCGATCCATAATGGCCAAAGATGGAA
TTCTGTTAGCCACAGGCTTGCATGTTCATCGCAGCAGTGCACACCAGGCAGGTGTGGGCA
CCATCTTTGACAGAGTTCTAACTGAACTAGTGGCCAAGATGAGAGAGATGAAGATGGAC
AAAACAGAACTGGGCTGTCTCAGGGCAGTTGTACTGTTTAACCCGGATGCTAAAGGGTTG
ACAGCAGTACAGGAAGTAGAACAGCTGCGTGAAAAAGTTTATGCCTCGTTAGAAGAATA
CACCAAAACCAGATATCCCGAAGAACCTGGGAGGTTTGCCAAACTTCTTCTACGACTGCC
TGCACTTAGGTCCATTGGCCTGAAGTGCCTGGAACATCTTTTCTTCTTTAAACTTATTGGTG
ACCAGCCTATCGACACTTTCCTCATGGAGATGCTAGAAAATCCAAGCCCAACGACATAG

Legend: eGFP-T2A-Neo-dT2A-LymRXR ligand sensor

Figure S1: The DNA sequence of the LymRXR sensor construct.
The LymRXR sensor construct is comprised of eGFP (green), T2A (blue), Neomycin (Neo) cassette (pink), degenerate T2A (blue) and GAL4 DNA binding domain fused to the LymRXR ligand binding domain (grey).

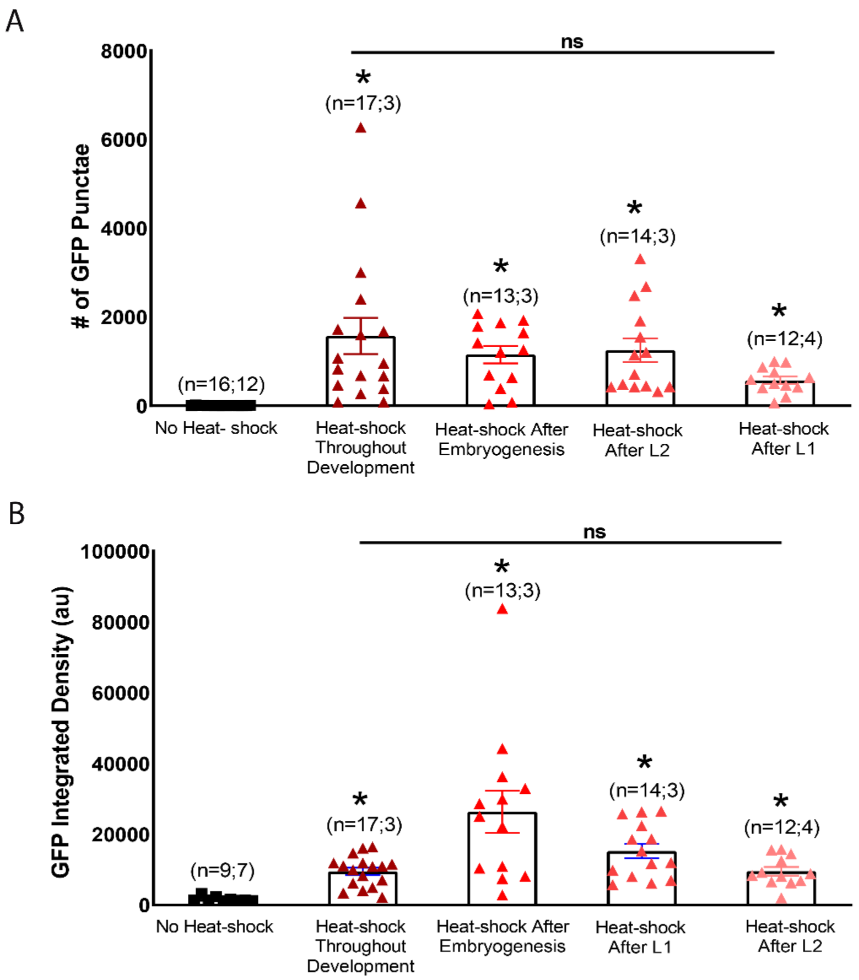

Figure S2: **Quantification of sensor expression following different heat-shock paradigms.** Quantitative analysis of GFP levels showing no significant changes in either the number of GFP punctae (A) or GFP integrated density (B) across larvae, heat-shocked at different developmental stages. All heat-shocked larvae exhibited significantly higher levels of GFP compared to control larvae not subjected to heat-shock. * $p < 0.05$; compared to no heat-shock controls; ns: no significant difference. Numbers in brackets represent the total number of CNS and the number of separate trials.
